# Figure-Associated Text Summarization and Evaluation

**DOI:** 10.1371/journal.pone.0115671

**Published:** 2015-02-02

**Authors:** Balaji Polepalli Ramesh, Ricky J. Sethi, Hong Yu

**Affiliations:** 1 Department of Quantitative Health Sciences, University of Massachusetts Medical School, Worcester, MA, United States of America; 2 School of Computer Science, University of Massachusetts, Amherst, MA, United States of America; 3 VA Central Western Massachusetts, Leeds, MA, United States of America; University of Vermont, UNITED STATES

## Abstract

Biomedical literature incorporates millions of figures, which are a rich and important knowledge resource for biomedical researchers. Scientists need access to the figures and the knowledge they represent in order to validate research findings and to generate new hypotheses. By themselves, these figures are nearly always incomprehensible to both humans and machines and their associated texts are therefore essential for full comprehension. The associated text of a figure, however, is scattered throughout its full-text article and contains redundant information content. In this paper, we report the continued development and evaluation of several figure summarization systems, the *FigSum+* systems, that automatically identify associated texts, remove redundant information, and generate a text summary for every figure in an article. Using a set of 94 annotated figures selected from 19 different journals, we conducted an intrinsic evaluation of *FigSum+*. We evaluate the performance by precision, recall, F1, and ROUGE scores. The best *FigSum+* system is based on an unsupervised method, achieving F1 score of 0.66 and ROUGE-1 score of 0.97. The annotated data is available at figshare.com (http://figshare.com/articles/Figure_Associated_Text_Summarization_and_Evaluation/858903).

## Introduction

Figures in biomedical publications are an essential part of biomedical knowledge. Futrelle [1] found that nearly 50% of article content in the biological domain is figure related. Figures assist researchers by providing evidence to support their finding, report their discovery, and generate new research hypotheses. On the other hand, hundreds of millions of figures are available in biomedical literature, which makes it difficult for biomedical researchers to search for figures. Therefore, we are developing an intelligent figure search engine (http://figuresearch.askhermes.org). Currently our figure search engine is available as a SciVerse API and has indexed over 4 million full-text biomedical journal articles published by Elsevier.

Given the enormous number of figures in biomedical literature, a key aspect in building an effective figure search engine is the ability to automatically interpret figure content. A number of studies have examined various approaches for the analysis and retrieval of relevant figures from literature [2–13]. The ImageCLEF (http://www.imageclef.org/) competition for automatic annotation and retrieval of images from literature has been held annually for the last 10 years. But, there is very limited research on extracting information related to figures from the full paper text in the biomedical domain [14]. Demner-Fushman [15] emphasized the importance of analyzing the text associated with the figure for its comprehension.

**Figure 1:**
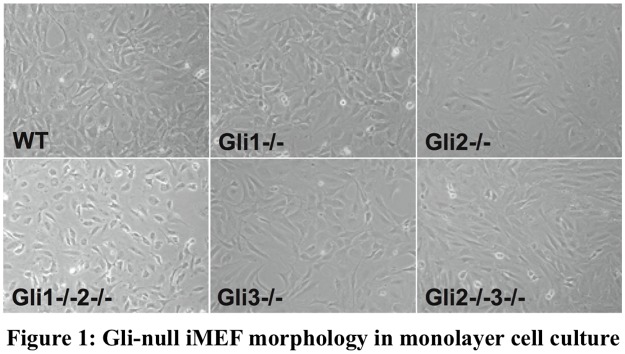
A sample figure with its caption. Fig. 1 appearing in article [18].

Our initial evaluation [16] showed that for a figure to be comprehended, it must be interpreted in conjunction with the text that refers to it in the article. We evaluated figure comprehension when a figure was presented (1) with its caption only, (2) with its caption along with the article title and abstract, and (3) with the article full text. The study found that presentation of the figure to biomedical researchers with just the title and abstract failed to convey 30% of the information related to the figure, compared to comprehension of the figure with the full text article. For example, Fig. 1 shows a figure along with its caption. The caption information alone is not sufficient for complete comprehension of the figure. Hence, the associated text from the full-text of the article is required to completely understand figures [17]. However, the associated text can be scattered throughout the full-text article and, moreover, can be redundant [16].

We therefore developed a preliminary figure summarization system called *FigSum* [14] that automatically generates a summary for every figure by extracting summary sentences from a full-text article. *FigSum* selects sentences to be included in the summary based on word-level similarities between the sentences and figure captions. A pilot evaluation showed biologists prefer the generated summaries [19], as they provide users with a new way for comprehending figure content without spending time navigating through the full-text article.

In our previous work we did not explore and evaluate other text summarization approaches. In this study, we compare and evaluate several summarization approaches, which we implemented as *FigSum+* systems:

1. The baseline *FigSum* approach, which is an information retrieval (IR) based approach wherein we find the sentences associated with a figure by finding sentences that are most similar to the figure caption.
2. The surface-cue approach, in which we generate a figure summary by identifying sentences and paragraphs that explicitly refer the figure.
3. A hybrid approach, in which we first identify paragraphs that explicitly refer the figure using the surface-cue approach and then we rank sentences by the centroid-based summarization algorithm.

**Figure 2:**
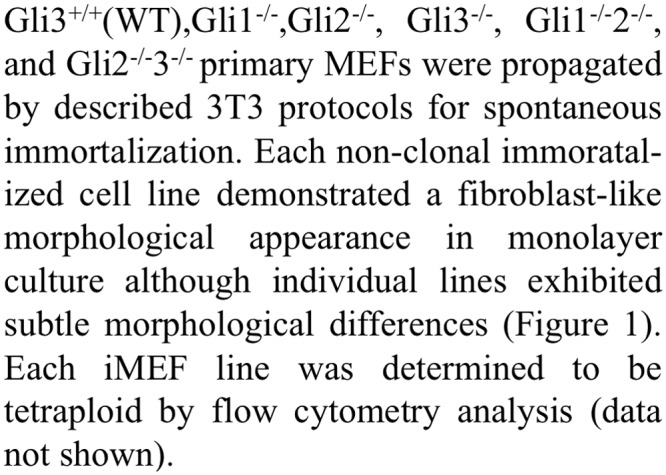
The summary generated by our system for figure shown in Fig. 1.

We perform intrinsic evaluations of these summarization approaches and report their performance. Fig. 2 shows the summary generated by our *FigSum+* summarization system using a surface-cue based approach for the figure shown in Fig. 1. The summary helps users better understand the figure. The summarization system also has the potential of improving figure retrieval and mining knowledge from figures.

## Related Work

Summarization is one of the most extensively studied fields in natural language processing (NLP). The summarization approaches can be broadly classified as extractive and abstractive [20, 21]. Extractive approaches extract and concatenate sentences from a text corpus to construct a summary, whereas abstractive summarization relies on natural language generation approaches that build new sentences representing the content of a text corpus to be summarized. In this work, we focus on the task of extractive summarization based on the text associated with a biomedical figure. The following sections review related work in open-domain text summarization, text summarization in the biomedical domain, and figure summarization.

### Open-Domain Summarization

Extractive summarization identifies sentences/paragraphs that subsume the key points of a text or a collection of texts. An early work by Luhn [22] proposed a simple idea based on the intuition that words occurring frequently in a document tend to describe the main topic and therefore sentences containing those frequent words shall be selected. Later studies improved this strategy by adding weight to words, using different techniques [23–27]. For example, Brunn et al. [23] used syntactic parsing to identify important words for summarization. Approaches that identify summary sentences based on location or other structural characteristics were also developed. For example, Nakov et al. [28] used citance (text that surrounds a citation reference) to summarize a document.

Edmundson [29] applied a linear function that combines different factors, including resemblance to the title, indicative context cues (e.g., *in summary*), keywords, and sentence location. Myaeng and Jang [30] extended this work by adding centrality of the sentence to the document to select summary sentences.

Later studies explored various information retrieval (IR) techniques, such as the TF × IDF weighting scheme, which alleviates the negative impact of overweighting of some common words [24, 31–33], and latent semantic analysis, which derives an implicit representation of text semantics based on observed word co-occurrences for summarization [34, 35]. For instance, Hovy and Lin [24] developed SUMMARIST, which integrates IR approaches, topic signatures (words that are highly descriptive of a document), dictionaries, and semantic knowledge derived from WordNet [36] to generate a summary. Inspired by link analyses and page rank algorithms for Web document retrieval, Mihalcea et al. [37] and Erkan et al. [38] applied a graph-based ranking method to select important sentences based on the graph derived from words and sentences. Radev et al. [39] developed a MEAD summarizer that generates summaries based on a cluster centroid calculated by TF × IDF word similarity.

Studies also explored supervised machine learning approaches for summarization [25], [40–43]. Kupiec et al. [25] developed a Naïve Bayes classifier using the following five features to select summary sentences from 188 documents: (1) length of the sentence, (2) occurrence of common phrases (such as “In conclusion”) or phrases appearing after sections such as “results” and “discussion”, (3) location of the paragraph in the document, (4) occurrence of high frequency words as in [22], and (5) sentences containing proper nouns and acronyms. Wang et al. [40] and Hirao et al. [41] ranked sentences using a support vector machine classifier to generate summaries. Leskovec et al. [43] built semantic graphs to extract subject–object–predicate triplets from sentences and then trained a support vector machine classifier to extract salient sentence triplets for summarization.

Evaluation is important in all NLP tasks. Mani [44] discussed various summarization evaluation criteria, including coherence, informativeness, relative utility, and relevance of the summary. Evaluation methods include word similarity measures such as cosine similarity [45], the overlap of a sequence of words that include *n*-grams (sequences of *n* number of word tokens) and longest common subsequence [46, 47], and the Bleu [48] machine translation evaluation measure for summarization [49]. The Document Understanding Conference (DUC) adopted the ROUGE package for content-based evaluation [50]. Among various summarization evaluation metrics[51, 52], ROUGE score is widely used and is calculated based on *n*-gram overlap between the gold standard and the summary generated. The scores range between zero and one, with a higher score indicating a summary closer to the gold standard. In our study we apply ROUGE to evaluate the quality of the summary generated by our system by comparing it to the gold standard.

### Biomedical Summarization

Open-domain summarization approaches are based on similarity and term occurrence approaches and would not be the optimal choice for biomedical text due to domain-specific characteristics. Biomedical summarization systems are frequently built upon biomedical knowledge resources, including the Medical Subject Headings (MeSH), the Unified Medical Language System (UMLS), and the Gene Ontology (GO) project, to overcome the challenge of domain-specific jargons.

Chiang, et al. [53] developed GeneLibrarian, which generates a viewgraph of genes related to the input query based on GO similarity. The system also generates a summary of a gene by selecting sentences based on term occurrences. Ling, et al. [54] developed approaches to automatically generate a structured gene summary by first retrieving gene-related documents and then extracting sentences containing factual information about the target gene. Jin, et al. [55] developed a query-based gene summarization system that integrates the page rank algorithm, sentence similarity, and the function of the gene represented by GO.

Many studies focused on summarizing the content in biomedical text using semantic resources. Bhattacharya, et al. [56] developed a method that computes similarities between the MeSH terms assigned to an article in addition to its word tokens and then returns the top N-ranked sentences as summary sentences. Plaza [57] generated summaries based on where the sentence resides. For example, the first few sentences are typical summary sentences. Reeve, et al. [58] developed the BioChain system using the concept chaining technique which links semantically related concepts in text using the UMLS [59]. Sentences with strong concept chains (where strength is based on the number of concepts) are used to form the summary. Fiszman, et al. [60] applied hand-crafted transformation rules to the output of SemRep (http://semrep.nlm.nih.gov/) to summarize content. SemRep is a system that extracts biomedical concepts and relations relevant to a given query from the MEDLINE records. Workman, et al. [61] later modified this work to generate domain-specific summaries to support database curation. Workman and Hurdle [62] applied SemRep to citations obtained from PubMed. They analyzed the outputs using statistical methods to automatically identify salient data in bibliographic text for summarization. Shang et al. [63] extended the work of Fiszman, et al. [60] to develop a multi-document summarizer for a given biomedical concept. Concepts and relations in sentences are extracted using SemRep. The sentences that contain high-frequency relations are then extracted as a summary. Other studies [64, 65] explored knowledge from the UMLS to construct a graph and then selecting summary sentences based on node clustering.

### Figure Summarization

Futrelle [66] proposed the idea of figure summarization. He described the challenges related to summarizing figures and emphasized the importance of captions and referring text. Bhatia and Mitra [67] applied a supervised approach to summarize document objects such as figures, tables and algorithms on a set of 290 document elements. Wu and Carberry [68] identified relevant paragraphs for images in news domain articles.

We developed a preliminary summarization system, *FigSum* [14], for the biomedical domain. *FigSum* first classifies sentences into the introduction, methods, results, and discussion categories using a supervised machine learning classifier [69]. Each sentence is then scored based on its TF × IDF weighted cosine similarity with the figure caption and the article’s central theme. The top-scoring sentence in each category is included in the summary. The *FigSum* system is integrated into our larger figure search system (http://figuresearch.askhermes.org). An online survey revealed that 65.2% participants found that *FigSum* summaries improved figure comprehension [19]. The current study explores additional figure summarization methods and performs an intrinsic evaluation to compare the performance of all systems.

## Methods

We explored several different summarization systems in which we explored different features. In the following, we first describe features and then systems.

### Features used for Summarization

We explored a number of features to build figure summarization systems.

**Figure 3:**
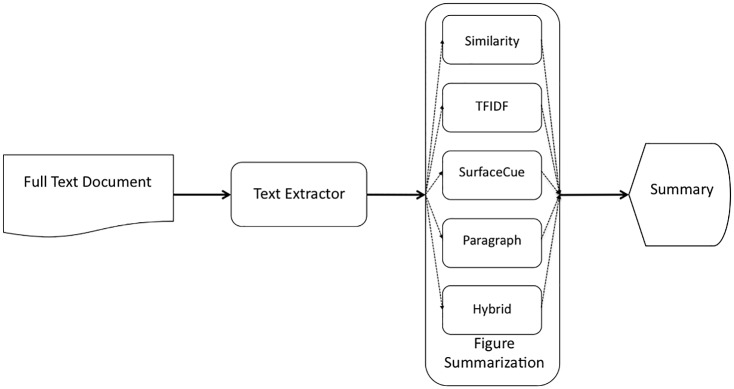
The general pipeline of our unsupervised *FigSum+* systems. Each implementation of the *FigSum*+ system differs by including only one of the five modules described in Section 3.2.1 and shown in the Figure Summarization component above: *Similarity*, *TFIDF*, *SurfaceCue*, *Paragraph*, or *Hybrid*.

1) IR based features
  a) Caption similarity feature—The cosine similarity value between each of the candidate sentences in the full text and the figure caption.
  b) Title similarity feature—The cosine similarity between each of the candidate sentences in the full text and the article title.
  c) Reference sentence similarity feature—The cosine similarity between each of the candidate sentences and sentences referring to the figure.
  d) TFIDF feature—The text association between each of the candidate sentences in the full text and the figure caption is computed by calculating the TF × IDF vector for every candidate sentence and figure caption. A score is calculated as the cosine similarity of the TF × IDF vectors of candidate sentences and the figure caption.
2) Reference Features
  a) Figure reference sentence feature—This feature represents if the sentence is figure referring (i.e., a sentence that incorporates figure reference cues such as *Fig. X*).
  b) Figure reference paragraph feature—This feature represents if the sentence belongs to the paragraph referring to the figure.
3) Hybrid feature—We first identify paragraphs in the full text article that contain figure reference sentences. We apply MEAD [39], a centroid-based text summarizer as described earlier on these sentences that are a part of the figure referring paragraphs. The *n* top scoring sentences are selected as summary sentences.
4) Position
  a) Distance from start feature—The position of the sentence from the start of the article.
  b) Distance from end feature—The position of the sentence from the end of the article.
  c) Distance from reference sentence feature—This is a binary feature that indicates if the candidate sentence is within 10 sentences of the reference sentence.
5) Sentence length feature—The length of the sentence.
6) Cue words and phrase feature—Authors of articles use certain cue words and phrases to describe document elements such as figures, as discussed in [67]. We use the list of 140 cue words and phrases listed in [67] and add the presence or absence of these cue words in the sentence as a binary feature.

### Figure Summarization Systems

In this section we describe a total of 23 figure summarization systems, which include our unsupervised *FigSum+* methods, and other unsupervised and supervised systems we built for comparison with our system.

***FigSum+* Systems**. Fig. 3 shows the general pipeline of the unsupervised *FigSum+* systems. Given a full text article, the Text Extractor module extracts individual sentences from the article. If the article is in XML file format, an XML parser module will process the text to extract sentences from the XML file. If the article is in PDF format, the PDF to text converter (PDFTextStream—http://snowtide.com) tool extracts the text from the PDF document and then we split the text to individual sentences using an in-house sentence splitter, which splits sentences by determining sentence boundaries such as period. The figure summarization module utilizes five unsupervised techniques, as described below, to summarize figures in the article and generate a summary for each figure.

We describe five different implementations of our unsupervised *FigSum+* systems, which differ on the features used in the figure summarization module. Each implementation of the *FigSum+* system differs by including one of the following five figure summarization modules: 1a, 1b, 2a, 2b, or 3.

(1) IR-based approaches: We explore two IR-based approaches for summarization.
  (a) *Similarity*—We select the top scoring sentences for the caption similarity feature as figure summary.
  (b) *TFIDF*—We select the top scoring sentences for the TFIDF feature as figure summary.
(2) Surface-cue approaches: We identify summary content using surface cues.
  (a) *SurfaceCue*—We use the figure reference sentence feature. It extracts all figure referring sentences in the full text as figure summary.
  (b) *Paragraph*—We use the figure reference paragraph feature. It extracts all paragraphs containing figure referring sentences as figure summary.
(3) *Hybrid*—We select the top scoring sentences for the hybrid feature as figure summary.

**Unsupervised baseline systems**. For comparison, we built three additional unsupervised systems as the baseline systems: *RandomSent*, *RandomPara* and *MEAD*. The *RandomSent* system randomly selects *n* sentences from the article as the summary for the figure. The *RandomPara* system randomly selects *n* paragraphs and then includes the first sentence of every randomly selected paragraph as the summary for the figure. For the last baseline system, *MEAD*, we applied the centroid summarizer MEAD to the entire full text article and select *n* top scoring sentences as the summary for each figure.

**Supervised baseline systems**. In *FigSum+*, we use five features as described in section 3.2.1; namely: caption similarity feature, TFIDF feature, figure reference sentence feature, figure reference paragraph feature and hybrid feature. We explored each of these features individually and trained baseline supervised machine learning models to generate figure summaries for evaluation. Each individual feature was used with both a naïve bayes (NB) and Support Vector Machine (SVM) classifier, thus resulting in 10 baseline supervised systems: *NBSimilarity*, *NBTFIDF*, *NBSurfaceCue*, *NBParagraph*, *NBHybrid*, *SVMSimilarity*, *SVMTFIDF*, *SVMSurfaceCue*, *SVMParagraph* and *SVMHybrid*.

**Unsupervised state-of-the-art system**. We also implemented the state of the art unsupervised system, *FigSum*, which summarizes the figure as described earlier, for performance comparison with *FigSum+*.

**Supervised state-of-the-art system**. We implemented the state-of-the-art system described in [67] by building two systems, *NBSOTA* and *SVMSOTA*, using the NB and SVM models respectively, with the features described in [67]. The features used are: figure reference sentence, figure reference paragraph, caption similarity, reference sentence similarity, distance from reference sentence and cue words.

We then extended the state-of-the-art system and build two more systems, *NBSOTA+* and *SVMSOTA+* using NB and SVM respectively, that incorporate all the features described in Section 3.1.

### Evaluation Metrics

Since the datasets are of different sizes, we calculate the micro-average of recall (R), precision (P), and F1 (F) scores to evaluate the summaries generated by each of the figure summarization systems described in Section 3.2. Recall is defined as the ratio of the number of sentences correctly identified by the system to the total number of sentences in the gold standard, precision is defined as the ratio of the number of sentences correctly identified by the system to the total number of sentences identified by the system, and the F1 score is the harmonic mean of recall and precision:

$$\text{Recall}=\frac{\#\;\text{of}\;\text{sentences}\;\text{correctly}\;\text{identified}\;\text{by}\;\text{the}\;\text{system}}{\text{Total}\;\#\;\text{of}\;\text{sentences}\;\text{in}\;\text{the}\;\text{gold}\;\text{standard}}$$ (1)

$$\text{Precision}=\frac{\#\;\text{of}\;\text{sentences}\;\text{correctly}\;\text{identified}\;\text{by}\;\text{the}\;\text{system}}{\text{Total}\;\#\;\text{of}\;\text{sentences}\;\text{identified}\;\text{by}\;\text{the}\;\text{system}}$$ (2)

$$\text{F}1\text{Score}=\frac{\left(2\;\times \;\text{Precision}\;\times \text{Recall}\right)}{\left(\text{Precision}\;+\;\text{Recall}\right)}$$ (3)

We also compute the ROUGE score using the parameters established by DUC 2007 [70]. Eq (4) gives the formula to calculate ROUGE-N, where *n* stands for the length of the *n*-gram, *gram_n_*, and *Count_match_(gram_n_)* is the maximum number of *n*-grams co-occurring in a candidate summary and a set of reference summaries. For every sentence in the summary generated by the *FigSum+* implementation, we calculate the ROUGE score against every sentence in the gold standard using the formula in Eq (4) and retain the best scores. Then we calculate the average of the best ROUGE score sentences for every figure: ROUGE-1 (R1) compares summaries based on the co-occurrence of unigrams (single words), ROUGE-2 (R2) compares summaries based on the co-occurrence of bigrams (two consecutive words), and ROUGE-SU4 (RSU4) compares summaries based on the co-occurrence of skip bigrams with a maximum gap length of four [50].

$$\text{ROUGE}-\text{N}=\frac{\sum_{S\in \{\text{ReferenceSummaries}\}}\sum_{gram_{n}\in S}Count_{match}(gram_{n})}{\sum_{S\in \{\text{ReferenceSummaries}\}}\sum_{gram_{n}\in S}Count(gram_{n})}$$ (4)

### Evaluation Data

We evaluated all the systems we built (*FigSum+*, baseline, and state-of-the-art) on a set of 19 full-text biomedical articles. Nine articles were randomly selected from our BioDRB corpus, a collection of 24 GENIA full-text articles fully annotated by us for discourse connectives and relations [71]. Four biologists with expertise in the biology domain each selected either two or three additional articles from various biomedical journals, for a total of 10 additional articles. The combined dataset of 19 articles comprises 94 figures and is made publicly available on figshare.com. The five *FigSum+* implementations are evaluated against the following two gold standards developed on these full-text articles; we selected two gold standards built using different approaches to show the robustness and efficacy of the five different techniques for figure summarization:

a) *FigSumGS1* dataset—A gold standard of 94 figures from 19 articles from various biomedical journals was created as follows: four biologists (B_1_—B_4_) read two papers each, for a sub-total of 8 articles, and then selected sentences within each article that summarized figure content. In addition, two (B_1_ and B_2_) of the four biologists, read and selected sentences from 11 additional articles, thus yielding a total of 19 articles in the gold standard. The two biologists (B_1_ and B_2_) identified 303 and 383 sentences, respectively. They had an inter-annotator agreement (IAA) of 0.68 Cohen’s κ value on the subset of 11 articles, which indicates a fair agreement between the annotators. The gold standard consists of a total of 678 sentences from 19 articles with a micro average of 7.21 sentences per figure and a macro average of 7.73 sentences per figure.
b) *FigSumGS2* dataset—A second gold standard consisting of a subset of 17 articles from the 19 articles collected in (a) was created using the guideline that was developed to evaluate the *FigSum* system [14]. Seven annotators with advanced degrees (MS and above) selected three to four sentences that best described the background of the figure, the methods used to generate the figure, the outcome of the figure, and the conclusion inferred from the figure on the subset of 17 articles consisting of 84 figures; this subset was chosen from the 19 articles due to constraints of manual annotation. Hence, for each figure, a summary consisting of 12 to 16 sentences was obtained. All seven annotators together identified 869 unique sentences from the 17 articles with a micro average of 10.34 unique sentences per figure and a macro average of 10.44 unique sentences per figure.

Table 1 shows the number of sentences and figures that appear in each article, the average number of unique sentences selected per figure, and the total number of sentences annotated for both gold standards.

**Table 1:** Statistics of the *FigSumGS1* and *FigSumGS2* gold standard datasets.

**Article**	**# of sents**	**# of figs**	**FigSumGS1 Dataset**		**FigSumGS2 Dataset**	
			**Avg # of unique sents per fig**	**# of sents annotated**	**Avg # of unique sents per fig**	**# of sents annotated**
1	190	3	5.0	15	11.7	35
2	144	3	18.0	54	11.7	35
3	173	7	5.0	35	8.0	56
4	160	5	8.6	43	10.2	51
5	172	4	12.8	51	10.5	42
6	140	5	8.4	42	10.8	54
7	281	9	7.8	70	11.8	106
8	137	9	4.7	42	6.3	57
9	142	5	6.2	31	11.2	56
10	87	5	6.4	32	8.4	42
11	162	6	6.0	36	9.7	58
12	34	2	7.5	15	6.0	12
13	50	3	8.0	24	11.0	33
14	138	3	5.0	15	12.7	38
15	119	3	12.3	37	11.0	33
16	120	5	9.2	46	12.4	62
17	152	7	5.1	36	14.1	99
18	157	4	6.2	25	-	-
19	184	6	4.8	29	-	-

## Results

We conducted an intrinsic evaluation to compare the performance of all five *FigSum+* implementations against baseline and state of the art unsupervised and supervised systems. Table 2 and Table 3 show the average performance of the various systems we built for summarization on the *FigSumGS1* and *FigSumGS2* datasets respectively. We chose the value of top *n* to be equal to the average number of sentences per figure in the gold standard. Hence, *n* is equal to 8 and 11 sentences per figure for *FigSumGS1* and *FigSumGS2* datasets respectively.

**Table 2:** Average performance and ROUGE scores (average ± standard deviation) of figure summarization techniques on *FigSumGS1* dataset.

		**System**	**Precision**	**Recall**	**F1 score**	**R1**	**R2**	**RSU4**
**Baseline State-of-the-art**	**Unsupervised**	**RandomSent**	0.06±0.09	0.06±0.12	0.06±0.09	0.28±0.09	0.11±0.10	0.13±0.09
		**RandomPara**	0.04±0.18	0.01±0.05	0.01±0.05	0.22±0.16	0.07±0.18	0.08±0.17
		**MEAD**	0.05±0.09	0.06±0.11	0.05±0.08	0.30±0.08	0.12±0.09	0.14±0.09
	**Supervised**	**NBSimilarity**	0.48±0.18	0.15±0.12	0.20±0.12	0.50±0.32	0.40±0.31	0.40±0.31
		**SVMSimilarity**	-	-	-	-	-	-
		**NBTFIDF**	-	-	-	-	-	-
		**SVMTFIDF**	-	-	-	-	-	-
		**NBSurfaceCue**	0.44±0.11	0.17±0.20	0.18±0.15	0.57±0.19	0.45±0.24	0.46±0.24
		**SVMSurfaceCue**	-	-	-	-	-	-
		**NBParagraph**	0.54±0.20	0.74±0.19	0.59±0.14	0.73±0.20	0.66±0.25	0.66±0.25
		**SVMParagraph**	-	-	-	-	-	-
		**NBHybrid**	0.80±0.19	0.37±0.15	0.49±0.15	0.95±0.13	0.94±0.17	0.94±0.17
		**SVMHybrid**	0.80±0.19	0.37±0.15	0.49±0.15	0.95±0.13	0.94±0.17	0.94±0.17
**State-of-the-art**	**Unsupervised**	**FigSum**	0.28±0.24	0.19±0.19	0.22±0.19	0.51±0.18	0.36±0.22	0.37±0.21
	**Supervised**	**NBSOTA**	0.44±0.15	0.74±0.17	0.53±0.12	0.63±0.12	0.53±0.15	0.53±0.14
		**SVMSOTA**	0.58±0.15	0.17±0.20	0.23±0.22	0.41±0.47	0.39±0.47	0.39±0.47
		**NBSOTA+**	0.47±0.16	0.70±0.19	0.53±0.13	0.67±0.16	0.57±0.20	0.57±0.20
		**SVMSOTA+**	0.78±0.17	0.34±0.14	0.47±0.14	0.95±0.14	0.93±0.18	0.93±0.18
**Our System**	**FigSum+**	**Similarity**	0.28±0.20	0.38±0.28	0.30±0.20	0.52±0.17	0.38±0.20	0.38±0.20
		**TFIDF**	0.30±0.25	0.34±0.24	0.30±0.22	0.51±0.21	0.38±0.25	0.38±0.24
		**SurfaceCue**	**0.96±0.13**	0.41±0.22	0.54±0.21	**0.97±0.07**	**0.97±0.10**	**0.97±0.10**
		**Paragraph**	0.64±0.27	**0.82±0.23**	**0.66±0.20**	0.74±0.20	0.67±0.25	0.68±0.24
		**Hybrid**	0.67±0.28	0.64±0.27	0.62±0.24	0.77±0.19	0.71±0.25	0.71±0.24

**Bold** indicates the best performance.

**Table 3:** Average performance and ROUGE scores (average ± standard deviation) of figure summarization techniques on *FigSumGS2* dataset.

		**System**	**Precision**	**Recall**	**F1 score**	**R1**	**R2**	**RSU4**
**Baseline State-of-the-art**	**Unsupervised**	**RandomSent**	0.08±0.08	0.09±0.11	0.08±0.09	0.32±0.08	0.15±0.09	0.16±0.08
		**RandomPara**	0.04±0.16	0.01±0.04	0.01±0.05	0.32±0.08	0.14±0.10	0.16±0.09
		**MEAD**	0.08±0.10	0.07±0.09	0.07±0.09	0.36±0.08	0.17±0.10	0.19±0.10
	**Supervised**	**NBSimilarity**	0.42±0.14	0.10±0.08	0.14±0.08	0.48±0.28	0.36±0.25	0.37±0.26
		**SVMSimilarity**	-	-	-	-	-	-
		**NBTFIDF**	-	-	-	-	-	-
		**SVMTFIDF**	-	-	-	-	-	-
		**NBSurfaceCues**	0.49±0.06	0.05±0.04	0.08±0.05	0.05±0.15	0.03±0.11	0.03±0.11
		**SVMSurfaceCue**	-	-	-	-	-	-
		**NBParagraph**	0.43±0.16	0.41±0.18	0.40±0.13	0.66±0.18	0.55±0.23	0.56±0.23
		**SVMParagraph**	-	-	-	-	-	-
		**NBHybrid**	0.55±0.17	0.18±0.08	0.26±0.11	0.75±0.25	0.66±0.33	0.66±0.33
		**SVMHybrid**	0.55±0.17	0.18±0.08	0.26±0.11	0.75±0.25	0.66±0.33	0.66±0.33
**State-of-the-art**	**Unsupervised**	**FigSum**	0.31±0.20	0.13±0.10	0.18±0.13	0.55±0.14	0.40±0.18	0.41±0.18
	**Supervised**	**NBSOTA**	0.37±0.14	**0.43±0.19**	0.38±0.13	0.59±0.11	0.46±0.13	0.47±0.13
		**SVMSOTA**	0.54±0.12	0.10±0.11	0.15±0.15	0.41±0.42	0.37±0.41	0.37±0.41
		**NBSOTA+**	0.37±0.13	**0.43±0.20**	0.38±0.13	0.60±0.15	0.47±0.18	0.47±0.18
		**SVMSOTA+**	0.54±0.16	0.18±0.12	0.26±0.11	**0.76±0.25**	0.67±0.33	0.67±0.33
**Our System**	**FigSum+**	**Similarity**	0.31±0.16	0.28±0.16	0.29±0.15	0.55±0.13	0.40±0.16	0.41±0.15
		**TFIDF**	0.27±0.22	0.20±0.14	0.22±0.16	0.51±0.18	0.36±0.22	0.36±0.21
		**SurfaceCue**	**0.63±0.36**	0.16±0.13	0.24±0.17	**0.76±0.24**	**0.68±0.32**	**0.68±0.31**
		**Paragraph**	0.51±0.24	0.42±0.22	**0.41±0.17**	0.66±0.18	0.56±0.22	0.56±0.22
		**Hybrid**	0.54±0.24	0.33±0.19	0.39±0.18	0.70±0.16	0.60±0.21	0.60±0.21

**Bold** indicates the best performance.

### Baseline Systems Result

For unsupervised baseline case, the *RandomSent* system had an F1 score performance of 0.06 and 0.08 and R1 scores of 0.28 and 0.32 on *FigSumGS1* and *FigSumGS2* datasets. The *RandomPara* system had an F1 score performance of 0.01 on both gold standards and R1 scores of 0.22 and 0.32 on *FigSumGS1* and *FigSumGS2* datasets respectively. The *MEAD* system achieved an F1 score performance of 0.05 and 0.07 and R1 scores of 0.30 and 0.36 on *FigSumGS1* and *FigSumGS2* datasets respectively. Whereas the state of the art unsupervised method *FigSum* system had an F1 score performance of 0.22 and 0.18 and R1 score of 0.51 and 0.55 on *FigSumGS1* and *FigSumGS2* datasets respectively.

For supervised baseline case, all the implementations of the baseline SVM systems, except for the system using the hybrid feature, failed to generate summaries. Both the NB and SVM based systems using the hybrid feature, *NBHybrid* and *SVMHybrid*, performed similarly and had the best baseline F1 score performance of 0.49 and 0.26 and R1 performance of 0.95 and 0.75 on the *FigSumGS1* and *FigSumGS2* datasets respectively.

### State-of-the-art Systems Result

For unsupervised state-of-the-art case, the unsupervised method *FigSum* system had an F1 score performance of 0.22 and 0.18 and R1 score of 0.51 and 0.55 on *FigSumGS1* and *FigSumGS2* datasets respectively.

For supervised state-of-the-art case, the NB-based supervised systems performed well compared to the SVM-based model similar to performance in article [67]. On *FigSumGS1* dataset, the NB-based state-of-the-art systems *NBSOTA* and *NBSOTA+* had an F1 score performance of 0.53 but *SVMSOTA+* achieved the second best R1 score of 0.95. Similarly, on *FigSumGS2* dataset, *NBSOTA* and *NBSOTA+* had the best F1 score performance of 0.38 and *SVMSOTA+* achieved the best R1 score of 0.76.

### Our FigSum+ Systems Result

The *SurfaceCue* implementation of *FigSum+* achieves the highest precision on both gold standards (0.96 and 0.63 on *FigSumGS1* and *FigSumGS2* datasets respectively) and the *Paragraph* implementation results in the highest recall (0.82 and 0.42 on *FigSumGS1* and *FigSumGS2* datasets respectively) and the highest F1 score (0.66 and 0.41 on *FigSumGS1 FigSumGS2* datasets respectively). The *Hybrid* implementation performs second best, yielding F1 scores of 0.62 and 0.39, respectively, on *FigSumGS1 and FigSumGS2* datasets.

The ROUGE score evaluation of *SurfaceCue* resulted in the highest R1, R2, and RSU4 scores, all above 0.97, on *FigSumGS1* dataset. Similarly, *SurfaceCue* resulted in the highest R1 score of 0.76 on *FigSumGS2* dataset.

## Discussion

In this study, we developed and investigated five implementations of *FigSum+* to automatically summarize every figure in a full-text biomedical article. Our summarization approaches remove redundant information by extracting sentences associated with the figure, reducing the redundancy and generating a succinct summary for every figure. We evaluated the performance of these approaches against two sets of gold standards. The first gold standard was comprised of 94 figures from 19 *PMC* articles (*FigSumGS1* dataset) and the second, a subset of 84 figures from 17 articles in the *FigSumGS1* dataset *(FigSumGS2* dataset). The *FigSumGS1* dataset showed a good IAA of 0.68 Cohen’s κ for a subset of 11 articles.

We first compared the performance of the five *FigSum+* systems against unsupervised baseline (*RandomSent, RandomPara* and *MEAD*) and unsupervised state-of-the-art (*FigSum*) systems. The improvement in both F1 score and ROUGE performance of *SurfaceCue*, *Paragraph, Hybrid* compared to all unsupervised systems was statistically significant (t-test, p < 0.05) on the *FigSumGS1* dataset. Whereas, for the *FigSumGS2* dataset comparison of unsupervised baseline systems, the improvement in the ROUGE score performance of *SurfaceCue*, *Paragraph* and *Hybrid* was statistically significant (t-test, p < 0.05) but the F1 score performance of only *Paragraph* and *Hybrid* was statistically significant (t-test, p < 0.05).

Supervised baseline systems using the same individual features as in the *FigSum+* systems were built using the NB and SVM machine learning models. All baseline SVM systems except for the system using the hybrid feature failed to generate figure summaries on both datasets. Among the supervised baseline systems based on NB, the system using the reference paragraph feature achieved an F1 score performance of 0.59 and 0.40 on *FigSumGS1* and *FigSumGS2* datasets respectively. The NB system using the hybrid feature had the highest R1 performance of 0.95 and 0.76 on *FigSumGS1* and *FigSumGS2* datasets respectively. The difference in F1 and ROUGE score performance of NB based systems was statistically significant over the *Paragraph* and *Hybrid* (t-test, p < 0.05).

We also compared the performance of the *FigSum+* systems against state-of-the-art supervised systems as described in section 3.2.5. The F1 score performance of the *Paragraph and Hybrid* systems were statistically significantly better than all state-of-the-art supervised systems (t-test, p < 0.05). In addition, the F1 score performance of *SurfaceCue* was statistically significantly better than the *SVMSOTA* system (t-test, p < 0.05) on *FigSumGS1* dataset. In terms of the ROUGE score performance, *SVMSOTA+* achieved the best scores using supervised approaches and the difference in performance against the best performing *SurfaceCue* was not statistically significant. The systems performed similarly on *FigSumGS2* dataset but the improvement of state-of-the-art supervised systems over *Paragraph and Hybrid* systems were not statistically significant.

The unsupervised *FigSum*+ systems performed better than the state-of-the-art supervised systems [67] (NBSOTA and SVMSOTA). Although this is an interesting result, previous studies have also demonstrated that unsupervised methods often have comparable, if not better, performance than supervised techniques [72–74]. In our case, this could be attributed to a number of reasons.

First, our systems were limited to the biomedical domain. Hence, these features could be better tuned to outperform in our domain. Second, although we used the same set of features as described in [67], the implementation of the similarity feature between our systems and [67] was different. We used the cosine similarity instead of the Okapi BM25 similarity, which we will explore in our future work. Third, the evaluation data used in [67] were different from the data used in our experiments.

We explored both supervised and unsupervised methods for figure summarization and concluded that the unsupervised techniques performed better. This is not surprising. Our annotated data size is small and therefore prone to the data sparseness challenge. One way to improve the performance is to increase the robustness of word representation. Word embedding [75] clusters similar words and therefore reduces the dimensionality of word features and may improve the performance of supervised learning.

As shown in Table 2 and 3, the feature of word similarity between the sentences to be included in a summary and the caption of a figure does not always improve the performance. This is not surprising. We found that frequently figure captions contain detailed methodological descriptions of experiments while summary sentences tend to interpret the results. This may explain why our first figure summarization *FigSum* does not perform as well as our *FigSum+* systems and why we need to explore additional features for optimal performance.

The *FigSum+* approaches *SurfaceCue, Paragraph*, and *Hybrid* had average F1 scores of 0.79 and 0.26, 0.84 and 0.27, and 0.82 and 0.21, respectively, for the *FigSumGS1* dataset and of 0.62 and 0.10, 0.62 and 0.24, and 0.64 and 0.21, respectively, for the *FigSumGS2* dataset. Human-generated summaries often show such variations as well [76, 77]. The performance differences of the various *FigSum+* techniques can be attributed to variations in the quality of the gold standard generated by the annotators.

Further analysis of the *FigSum+* performance on *FigSumGS1* dataset using Spearman Rank Correlation showed that there was no correlation between the F1 score and the length of the article or the number of figures. However, the F1 score of *SurfaceCue* showed moderate negative correlation (rho = -0.51, p < 0.05) with the average number of sentences per figure. For *FigSumGS2* dataset, the length of the article had a moderate negative correlation with the performance of *Paragraph* (rho = -0.52, p < 0.05) and *Hybrid* (rho = -0.50, p < 0.05) implementations and the average number of sentences per figure and had a negative correlation with the performance of *Paragraph* (rho = -0.71, p < 0.05) and the *Hybrid* (rho = -0.74, p < 0.05) implementations. This finding suggests that longer summaries tend to have lower quality.

The *SurfaceCue* system had a near perfect ROUGE score for *FigSumGS1* dataset, since the annotators picked figure-referring sentences as part of the gold standard. Although the *SurfaceCue* approach had a very high ROUGE score, it also had a very low recall (0.41 for *FigSumGS1* and 0.16 for *FigSumGS2* datasets) compared to the *Paragraph* and *Hybrid* approaches. There was no correlation between the ROUGE score performance and the length of the article, the number of figures, or the average number of sentences per figure for the *FigSumGS1* dataset. Similarly, there was no correlation between the number of figures or the average number of sentences per figure except length of the article, which had a negative correlation with *SurfaceCue* (rho = -0.72, p < 0.05) for *FigSumGS2* dataset.

The *FigSum+* approaches performed well against two different gold standards constructed using different criteria, demonstrating the robustness of the approaches and their efficacy in rendering comprehensive figure summaries. It was also interesting that one article in *FigSumGS1* dataset had an F1 score of 0.44 for the *Hybrid* approach but achieved an R1 score of 0.85, indicating that the quality of the summaries extracted by the *FigSum+* implementations were as good as human-generated summaries.

**Figure 4:**
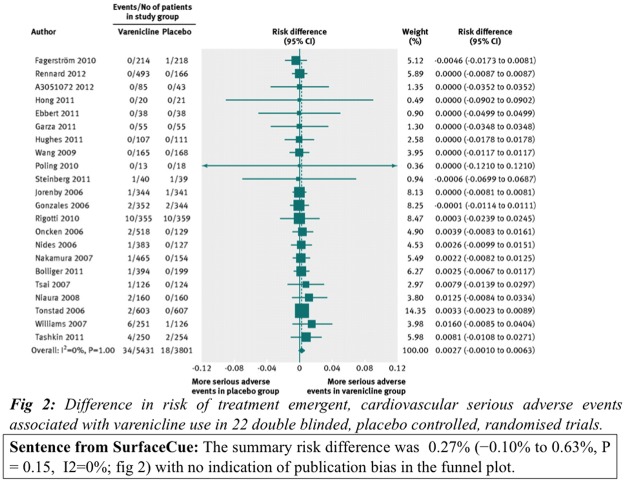
A sample figure with its caption and summary generated by *SurfaceCue*. Fig. 2 appearing in article [78].

**Figure 5:**
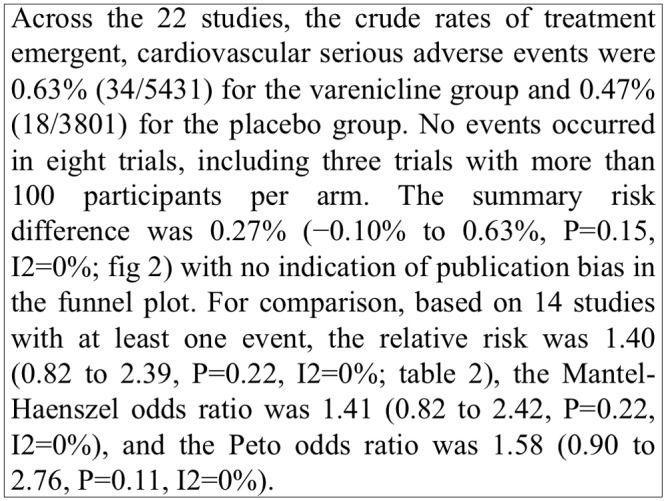
The summary generated by *Paragraph* methods for the figure in Fig. 4.

One of the inherent problems of extractive summaries is that they lack coherence and certain sentences do not make sense when taken out of context (e.g., as in the *SurfaceCue* implementation). For example, Fig. 4 shows a figure along with its caption and the sentence extracted by the *SurfaceCue* method. The sentence “*The summary risk difference was 0.27% (−0.10% to 0.63%, P = 0.15, I2 = 0%; fig. 2) with no indication of publication bias in the funnel plot*”, provides very little context for the figure. To overcome this problem, we extracted whole paragraphs where figure-referring sentences appeared, as in the *Paragraph* approach. Fig. 5 shows the summary extracted by the *Paragraph* method for the figure shown in Fig. 4. The summary provides more information and context to help understand the figure better. We believe this method provides users with the sentence context and improves the overall comprehension of the figure while reducing user information overload.

## Limitations and Future Work

There are, however, certain limitations to the study. The current results are based on only 94 figures from 19 biomedical articles. Although this number of figures is small, it is on a par with other studies that also require extensive manual annotation [67]. The results indicate that the *FigSum+* approaches—especially *Paragraph* and *Hybrid*—can generate summaries that are closely related to the information deemed important by experts to explain the content of figures. Our annotation may have limitations as well. Although biomedical experts annotated the gold standard summaries, we found all of whom selected sentences from paragraphs referenced a figure and bias may be introduced. A future work for creating a summary gold standard is to extract all relevant sentences and then to ask experts to pick out the most informative and representative ones. As stated earlier, in the future, we will explore class-based language modeling approaches (word embedding) to overcome the data sparseness challenge. We also evaluate the system’s utility by comparing it with other systems in an extrinsic evaluation.

## Conclusion

This study explored a number of supervised and unsupervised approaches to summarize figures in biomedical articles by aggregating sentences associated with a figure and removing redundant sentences. Our evaluation results show that a simple unsupervised *FigSum*+ system that is based on surface cues achieved the best F1 score of 0.66 and ROUGE-1 score of 0.97. *FigSum+* can be readily implemented with minimum computation cost and thereby maximizing its speed. These results demonstrate that the *FigSum+* approaches present a promising approach for figure summarization by reducing information overload while improving users’ information-seeking behavior and maintaining information content.

## Supporting Information

S1 Supplemental Material Annotation Guideline—Figure Summarization. (DOC)
