# Supplementary material for: Figure-Associated Text Summarization and Evaluation
Source: PLoS One. 2015 Feb 2;10(2):e0115671. doi: 10.1371/journal.pone.0115671 (PMC4313946; doi:10.1371/journal.pone.0115671)
Supplement: S1 Supplemental Material — (DOC) [file pone.0115671.s001.doc]

**Annotation Guideline – Figure Summarization**

**General Rules**

1. Any text that directly refers to a figure by indicating the figure number in parentheses or in the text itself must be associated with that figure (see example 1).
2. When multiple figures are referenced in text, associations are made between the text and all figures referenced (see example 2)
3. Text containing referents to the elements portrayed or described in the figure including data, treatments, methods, interpretations and materials used are associated with the figure.
4. Text Scope
   - In general the text scope includes the full paragraph in which a reference to an image is included as the associated text
   - In the Methods section, text associated with the entire method or process should be included as the associated text. This may involve multiple paragraphs or the entire set of paragraphs included under a heading for the method
5. The confidence level of the annotator in making the annotation should also be included. Indicate either high or low confidence as part of the annotation.

**Associations for Paper Sections**

References and text associated with figures differ according to the section of the paper in which they appear.

- In the Introduction section, in general the associated text can be identified by General Rule 1.
- In the Methods section, the associated text can be identified by General Rule 3. The following types of text are associated with a figure:
  - Materials or specimens labeled or referred to in charts and figures
  - Procedures used in preparation of subjects related to the data presented
  - Treatments or applications indicated
  - Experimental conditions shown or used as comparison points
  - Methods resulting in the data shown
  - Sampling points or treatment duration indicated
  - Statistical methods used to analyze data presented
- In the Results section typically the associated text can be identified by General Rule1.
- In Discussion, in certain cases, a figure is not directly referenced. In these cases, the authors sometimes use “visual” words indicating they are referencing a figure: ***show(ed), demonstrated, present(ed), illustrate(d)***. In referencing figures which show results, the authors often use such words as: ***resulted in, found (finding), confirmed, prove, concluded (conclusion).***

**Examples**

Following are examples of the general rules.

***Example 1 - General Rule 1***

**Associated Text**

Administration of the same dose of CGRP-(8—37) also markedly reduced toxin A-mediated increases in mucosal [3H]mannitol permeability (by 83%, *P* ,0.01; Fig. 2).

Fig. 2. Inhibition of toxin A-mediated mucosal mannitol permeability

by CGRP-(8—37). Experiments were performed as described in

Fig. 1. After 4 h, ileal loops were excised and intestinal permeability

to [3H]mannitol was estimated by scintillation counting of aliquots of

loop fluid. Results are means6SE from 7 loops. ††*P*,0.01 compared

with control; ***P* , 0.01 compared with toxinAalone.

***Example 2 - General Rule 2***

**Associated Text**

The principal finding of this study is that instillation of purified C. difficile toxin A into rat ileal loops induces an early increase in the CGRP content of dorsal root ganglia and intestinal mucosa.

**This single sentence is associated with 2 figures, Fig 5 and Fig 6**

Fig. 5. Effect of toxin A (TxA) on CGRP content of ileal mucosa. Rat ileal loops were formed and injected with 0.4 ml Tris buffer containing 5 μg purified *C. difficile* toxin A. After 30, 60, and 120 min, loops were harvested, and mucosal CGRP content was determined as described in MATERIALS AND METHODS. Results are means 6 SE from 5–6 loops. ***P* , 0.01 compared with control.

Fig. 6. Effect of toxin A (TxA) on CGRP content of lumbar dorsal root ganglia. Rat ileal loops were formed and injected with 0.4 ml Tris buffer containing 5 μg purified *C. difficile* toxin A. After 30, 60, and 120 min, lumbar dorsal root ganglia were harvested and CGRP content was determined as described in MATERIALS AND METHODS.

Results are means 6 SE from 5–10 loops. **P* , 0.05 compared with

control.

***Example 3- General Rule 3***

**Associated Text**

To determine the cell(s) of origin of increased CGRP production in ileal loops exposed to toxin A, immunohistochemical staining was performed using tissue sections from ileal loops exposed to toxin A

Fig. 7. CGRP immunohistochemistry in normal and toxin A-treated ileum. Rat ileal loops were formed and injected with 0.4 ml Tris buffer containing 5 μg purified *C. difficile* toxin A. After 60 and 120 min, loops were harvested andCGRP immunohistochemistry was performed as described in MATERIALS AND METHODS. In normal ileum CGRP

immunoreactivity is mainly associated with neurons present in the lamina propria and the submucosa (Fig. 7*A*). Nochange in ileal CGRP immunoreactivity was observed after treatment with toxin A for 1 h (data not shown).Instillation of toxin A into ileal loops for 2 h markedly increased CGRP immunoreactivity compared with control.Increased staining is particularly apparent in lamina propria neurons (Fig. 7*B*). Control ileal sections processed inparallel but incubated with rabbit nonimmune serum showed no immunostaining (Fig. 7*C*). Magnification, 3200.

Methods (immunohistochemistry), materials( ileal loops, CGRP) and treatments (Toxin A) indicated in the above text are associated with the presented results of the method and treatment using the stated materials in Figure 7.

***Example 4 – General Rule 3***

**Associated Text**

Furthermore, CGRP- (8—37), a CGRP inhibitor, almost completely abolished mucosal permeability to [3H]mannitol and substantially inhibited intestinal fluid secretion and histological damage induced by toxin A in rat ileum.

Fig. 3. Inhibition of toxin A-induced enteritis by CGRP-(8—37). Rat ileal loops were injected with 0.4 ml Tris buffer alone or with Tris buffer containing 5 μg purified *C. difficile* toxin A. Test animals were pretreated with either PBS or PBS containing CGRP-(8—37) (80 nmol/kg iv) 5 min before and 25, 55, and 85 min after toxin A administration. After 4 h, loops were removed, full-thickness samples were fixed in Formalin, and sections were stained with hematoxylin and eosin. *A*: buffer-treated ileal loop showing normal mucosa. *B*: ileal loop exposed to toxinA showing disruption of villus architecture, goblet cell discharge, and tissue necrosis. *C*: ileal loop pretreated with CGRP-(8—37) before challenge with toxin A, showing almost complete prevention of the intestinal effects of toxin A. Magnification, 3140

Materials (CGRP, ileum), treatment (ToxinA) and results (inhibition of intestinal effects) are shown in the figure and referred to in the text.

***Example 5 – General Rule 3***

Fig. 2. Inhibition of toxin A-mediated mucosal mannitol permeability by CGRP-(8—37). Experiments were performed as described in Fig. 1. After 4 h, ileal loops were excised and intestinal permeability to [3H]mannitol was estimated by scintillation counting of aliquots of

loop fluid. Results are means6SE from 7 loops. ††*P*,0.01 compared

with control; ***P* , 0.01 compared with toxinAalone.

**Associated Text**

Administration of the same dose of CGRP-(8—37) also markedly reduced toxin A-mediated increases in mucosal [3H]mannitol permeability (by 83%, *P* ,0.01)

Materials, treatment and data are shown in the figure and referred to in the text.

Multiple Methods paragraphs linked to single figure.

***Example 6 – General Rule 4***

**Associated Text**

***Measurement of mannitol permeability and fluid secretion in rat ileal loops****.* Two days before the experiment, rats were anesthetized by intraperitoneal injection of pentobarbital sodium, and polyethylene catheters (1.27 mm in diameter; Clay Adams, Parsippany, NJ) were placed in the right jugular vein and subcutaneously exteriorized in the intrascapular region. After implantation of the catheters, animals were kept under continuous observation until the day of the experiment.

Two days after surgery, fasted rats were anesthetized by intraperitoneal injection of pentobarbital sodium (40 mg/kg ip), and a laparotomy was performed. Both renal pedicles were ligated, and 10 µCi of [^3^H]mannitol were injected into the inferior vena cava. Two 5-cm closed loops were then formed in the distal ileum, with at least 5 cm distance separating each loop. Five minutes before instillation of toxin A [5 µg in 0.4 ml tris(hydroxymethyl)aminomethane (Tris) · HCl, pH 7.4] or buffer into ileal loops, rats were treated with an equal volume (40 µl) of either PBS or CGRP-(8
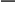
37) (80 nmol/kg body wt) administered via the jugular vein catheter. A similar dose of CGRP-(8
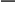
37) was found to significantly inhibit neurogenic vasodilation in rat paw skin after intraplantar injection of sodium nitroprusside ([17](http://ajpgi.physiology.org/cgi/content/full/274/1/G196#B17%23B17)). The same amount of PBS or CGRP-(8
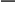
37) was also administered 25, 55, and 85 min after injection of toxin A. Multiple doses of CGRP-(8
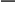
37) were given to minimize possible in vivo degradation of the antagonist over the course of the experiment. Animals were maintained under general anesthesia for the duration of the experiment with pentobarbital sodium (20 mg/kg ip) given ~20 min after the end of the operation. Animals were also placed on a heating pad to keep their body temperatures at 37-38°C. After 4 h, the animals were killed, the ileal loops were removed, and the weights and lengths were measured. Intestinal fluid secretion (weight-to-length ratio; mg/cm) and mucosal [^3^H]mannitol permeability [disintegrations per minute (dpm) per centimeter loop] were determined as described previously ([25](http://ajpgi.physiology.org/cgi/content/full/274/1/G196#B25%23B25)).

Fig. 1. Inhibition of toxin A-induced intestinal fluid secretion by calcitonin gene-related peptide-(8—37) [CGRP-(8—37)]. Rat ileal loops were injected with 0.4 ml Tris buffer alone or with Tris buffer

containing 5 μg purified *C. difficile* toxin A. Test animals were pretreated with either phosphate-buffered saline (PBS) or PBS containing CGRP-(8—37) (80 nmol/kg iv) 5 min before and 25, 55, and

85 min after toxin A administration. After 4 h, ileal loops were harvested and intestinal fluid secretion was estimated as the loop weight (mg)-to-length (cm) ratio. Results are means 6 SE from 7 loops. ††*P* , 0.01 compared with control; ***P* , 0.01 compared with

toxinAalone.
